# Note from Dr. Ashwell

**Published:** 1847-04

**Authors:** Samuel Ashwell

**Affiliations:** Grafton-street


### DR. ASHWELL'S TREATISE ON THE DISEASES OF WOMEN.

*To the Editor of the British and Foreign Medical Review.*

Sir,—Having read in the January Number of the Review your notice of my Second Edition, I beg your insertion of the subjoined statement.

You observed that there is an identity of the second and first impressions of my work 'On the Diseases of Women,' in all except the First Part—(208 pages.)

The explanation is a very simple one. The work appeared (for my convenience) in Parts, one thousand of each having been printed. Now, as a long time elapsed between the publication of the first and third portions, it was found, when the whole of the complete books had been sold, that there were left on hand about 250 copies of the third, and somewhat less of the second parts.

At this time, as there was not a copy in the trade, Mr. Highley suggested, to prevent the book being long out of print, that the first part, comprising 208 pages, should be rewritten, and by this means a small second edition would be obtained, and the demand for the work temporarily supplied. I beg to thank the profession for their good opinion of the book, and to assure them that no pains shall be spared to make it worthy their continued approval.

I am, Sir, yours respectfully,

Samuel Ashwell.

Grafton-street, March 22d, 1847.

